# Knockdown resistance mutations are common and widely distributed in *Xenopsylla cheopis* fleas that transmit plague in Madagascar

**DOI:** 10.1371/journal.pntd.0011401

**Published:** 2023-08-22

**Authors:** Shelby M. Hutton, Adelaide Miarinjara, Nathan E. Stone, Fara N. Raharimalala, Annick O. Raveloson, Ravo Rakotobe Harimanana, Mireille Harimalala, Soanandrasana Rahelinirina, Ryelan F. McDonough, Abbe D. Ames, Crystal Hepp, Minoarisoa Rajerison, Joseph D. Busch, David M. Wagner, Romain Girod

**Affiliations:** 1 Pathogen and Microbiome Institute, Northern Arizona University, Flagstaff, Arizona, United States of America; 2 Medical Entomology Unit, Institut Pasteur de Madagascar, Antananarivo, Madagascar; 3 Plague Unit, Institut Pasteur de Madagascar, Antananarivo, Madagascar; 4 Office of Field Operations, Food Safety Inspection Service, Department of Agriculture, Souderton, Pennsylvania, United States of America; CNRS: Centre National de la Recherche Scientifique, FRANCE

## Abstract

**Background:**

Plague, caused by the bacterium *Yersinia pestis*, remains an important disease in Madagascar, where the oriental rat flea, *Xenopsylla cheopis*, is a primary vector. To control fleas, synthetic pyrethroids (SPs) have been used for >20 years, resulting in resistance in many *X*. *cheopis* populations. The most common mechanisms of SP resistance are target site mutations in the voltage-gated sodium channel (VGSC) gene.

**Methodology/Principal findings:**

We obtained 25 collections of *X*. *cheopis* from 22 locations across Madagascar and performed phenotypic tests to determine resistance to deltamethrin, permethrin, and/or dichlorodiphenyltrichloroethane (DDT). Most populations were resistant to all these insecticides. We sequenced a 535 bp segment of the VGSC gene and identified two different mutations encoding distinct substitutions at amino acid position 1014, which is associated with knockdown resistance (*kdr*) to SPs in insects. *Kdr* mutation L1014F occurred in all 25 collections; a rarer mutation, L1014H, was found in 12 collections. There was a significant positive relationship between the frequency of *kdr* alleles and the proportion of individuals surviving exposure to deltamethrin. Phylogenetic comparisons of 12 VGSC alleles in Madagascar suggested resistant alleles arose from susceptible lineages at least three times. Because genotype can reasonably predict resistance phenotype, we developed a TaqMan PCR assay for the rapid detection of *kdr* resistance alleles.

**Conclusions/Significance:**

Our study provides new insights into VGSC mutations in Malagasy populations of *X*. *cheopis* and is the first to report a positive correlation between VGSC genotypes and SP resistance phenotypes in fleas. Widespread occurrence of these two SP resistance mutations in *X*. *cheopis* populations in Madagascar reduces the viability of these insecticides for flea control. However, the TaqMan assay described here facilitates rapid detection of *kdr* mutations to inform when use of these insecticides is still warranted to reduce transmission of plague.

## Author summary

In Madagascar, the Oriental rat flea *Xenopsylla cheopis* is the primary vector of *Yersinia pestis*, which causes plague. This flea has been reported to be resistant to insecticides used for vector control. The objective of this study was to examine the target site mutations in the voltage-gated sodium channel (VGSC) gene in *X*. *cheopis* populations sampled throughout the island and previously tested for synthetic pyrethroid (SPs) susceptibility. Two widespread mutations associated with knock-down resistance (*kdr*) to SPs were identified, and a significant relationship between the frequency of *kdr* alleles and the SP resistance phenotype was revealed. The results raise concerns about the overall efficacy of SPs for controlling *Yersinia pestis* transmission by *X*. *cheopis* in Madagascar.

## Introduction

Plague, a zoonotic disease caused by the bacterium *Yersinia pestis*, is one of the most important diseases in human history and is estimated to have killed hundreds of millions of people during three pandemics [1]. Despite its importance to humans, plague is primarily a disease of rodents that naturally cycles within rodent populations via flea vectors [2]. Most human cases are of the bubonic form, which is acquired via the bite of an infected flea. Without treatment, bubonic plague can progress to the more fatal septicemic and/or pneumonic forms [3]. Pneumonic plague is particularly dangerous because it has a high fatality rate without early treatment and can be transmitted from person to person via respiratory droplets [4,5]. The third plague pandemic, which began in the mid-1800s, led to the establishment of new plague foci worldwide, notably in Asia, the Americas, and Africa, including Madagascar [6]. Currently, Madagascar reports the majority of global human plague cases each year [7].

Plague was introduced to the port of Toamasina, Madagascar, in the late 1800s, likely via steamboats from India [8,9]; periodic introductions and outbreaks in seaport cities followed. In 1921, plague became established in the highlands and subsequently disappeared from the coasts, except for periodic outbreaks in the port cities of Mahajanga [10] and more recently, Toamasina [11]. The endemic flea vector, *Synopsyllus fonquerniei*, is thought to play an important role in maintaining plague in the environment because both it and human plague cases are primarily restricted to elevations of 800 meters or higher. Although *S*. *fonquerniei* is found on rodents in the environment, *X*. *cheopis* infests black rats (*Rattus rattus*) inside human dwellings and, therefore, is the primary plague vector that transmits the bacterium to humans in Madagascar [12]. Because there is currently no effective and safe plague vaccine available, insecticides, together with antimicrobial treatment of cases and chemoprophylaxis of contacts, are the foremost tool for responding to, and preventing, plague outbreaks in humans.

The first insecticide used for plague control in Madagascar was dichlorodiphenyltrichloroethane (DDT), applied as a powder inside households starting in 1947 [13,14]. Beginning in the 1960s, descriptions of DDT-resistant fleas began arising in many plague-endemic regions [15]. Despite this situation, DDT was used until the end of the 1980s, when the official insecticide used for plague control was switched to deltamethrin, a type of synthetic pyrethroid (SP) used worldwide for insect control [16]. Similar to DDT, the continuous use of deltamethrin for several decades has resulted in increasing levels of resistance to this insecticide in *X*. *cheopis*. Recent studies have shown that some *X*. *cheopis* populations are resistant to multiple insecticides, including DDT, deltamethrin, and other SPs, demonstrating the threat that insecticide resistance poses to the long-term efficacy of chemical control of flea vectors in Madagascar [17,18].

Resistance to SPs is often caused by genetic mutations in the voltage-gated sodium channel (VGSC) gene in arthropods. This gene encodes the VGSC protein that is responsible for propagating action potentials and transmitting nerve impulses along the nerve axon. When a pyrethroid compound binds to this channel it causes prolonged opening and disrupts nerve function, resulting in paralysis and death [19]. However, point mutations in the VGSC gene can alter amino acids in the protein and reduce the binding efficiency of an insecticide. This type of resistance is referred to as knockdown resistance (*kdr*) and is characterized by heritable insensitivity to all types of pyrethroids to varying degrees [19, 20]. Because mutations in the VGSC gene threaten the use of SPs for pest and vector control, they have been described in many arthropod species [21]. Some of the most reported resistance mutation sites occur in domain II segments 4–6, which contains an important binding site for pyrethroids [22,23].

Although mutations in the VGSC gene have been well-characterized in many insect species, very little is known about the relationship between these mutations and resistance to SPs in fleas. Understanding the genetic mechanisms that can lead to SP resistance in *X*. *cheopis* is important for assessing the effectiveness of this insecticide class for vector control in Madagascar. Previous studies have reported finding *kdr* mutations in the VGSC gene of *X*. *cheopis* in both Uganda [24] and China [25], suggesting a probable mechanism for SP resistance. Our study is the first to describe VGSC mutations in Madagascar and to investigate the genotype-phenotype relationship in *X*. *cheopis*.

## Methods

### Ethics statement

This study was carried out in accordance with the directive 2010/63/EU of the European Parliament (https://eur-lex.europa.eu/Lex%20UriServ/LexUriServ.do?uri=OJ%3AL%3A2010%3A276%3A0033%3A0079%3AEN%3APDF) regarding the protection of animals used for scientific purposes. Also, protocols involving field and laboratory animal handlings were approved by the animal ethics committee of the Institut Pasteur de Madagascar (Letter no.425/2021/IPM/DS/CEA).

### Flea collections and phenotype testing

**Fig 1:**
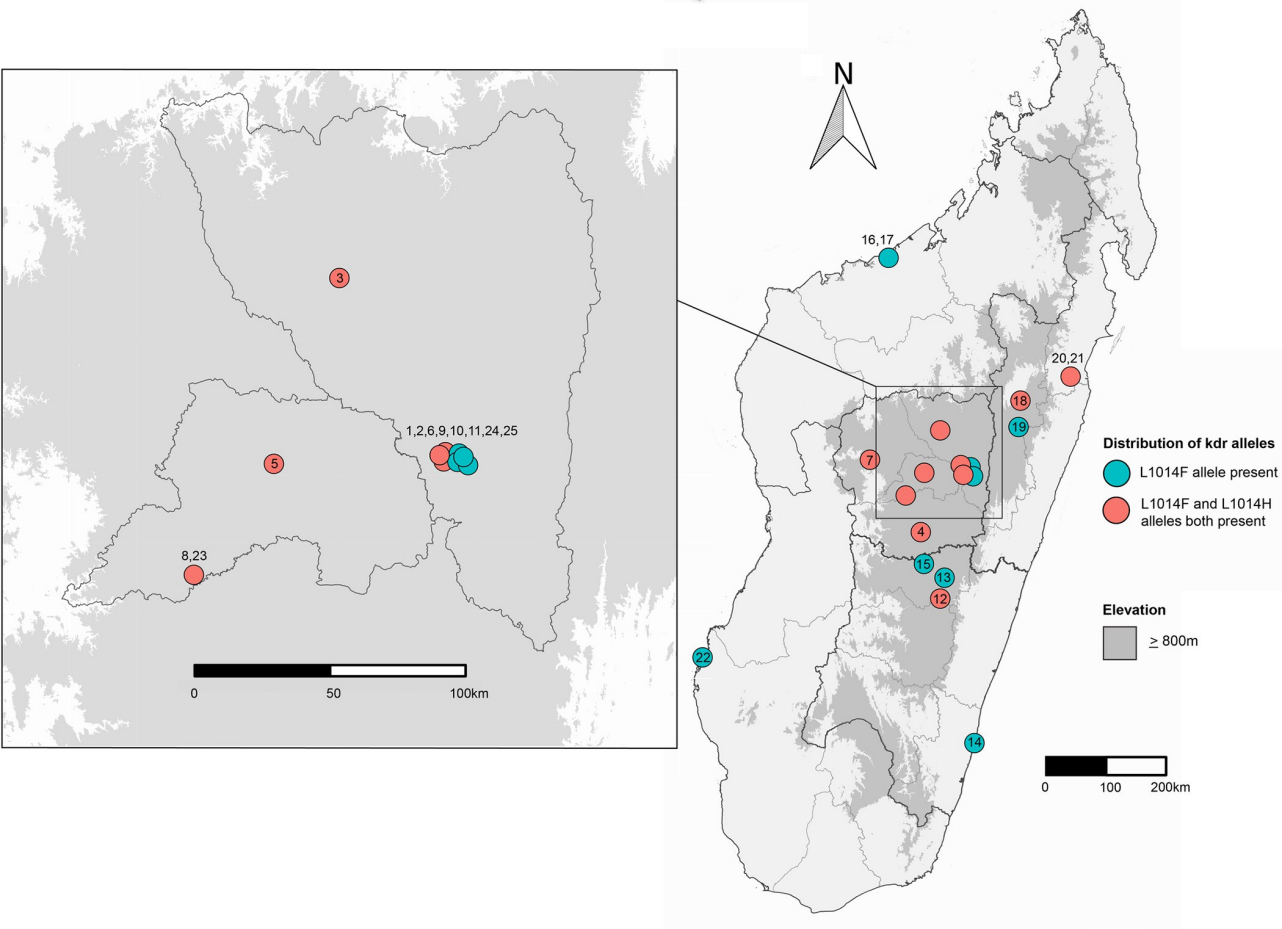
*Xenopsylla cheopis* flea sampling locations in Madagascar. Sampling locations, with assigned collection numbers, are indicated with colored circles. Colors correspond to the particular *kdr* mutation(s) present in some or all of the fleas collected at that location; mutation L1014H was only found at locations where mutation L1014F was also present. Sampling points with multiple collection numbers indicate collections made at the same location but in different years. Areas of ≥ 800 meters elevation are indicated with gray shading; plague-endemic regions in Madagascar are mostly limited to these higher elevations. This map was created using R Studio software by Team R [26]. Data used to create this map were sourced from GADM (https://gadm.org/data.html).

Fleas were collected from live black rats and other small mammals trapped during field investigations carried out in response to plague cases/clusters reported in Madagascar or during passive animal surveillance efforts from 2002 to 2020. We chose a subset of 25 *X*. *cheopis* collections from 22 locations for this genetic study (Fig 1 and Table 1). Small mammals were trapped in wire-mesh and Sherman traps, euthanized by cervical dislocation, and combed for fleas, which were then transported live to the Institut Pasteur de Madagascar (IPM) insectarium. The fleas morphologically identified as *X*. *cheopis* were reared under specific laboratory conditions until the collections reached adequate numbers for insecticide bioassays (Table 1). In most cases, fleas from field collections were not tested immediately but instead were used to establish lab colonies tested at a later generation. The full details of the rodent trapping, flea collection, and rearing methods have been described previously [16]. None of the lab colonies from this study were maintained under insecticide selection pressure; therefore, any selection from SP insecticides had occurred in free-living flea populations. Insecticide bioassays were performed following a protocol modified from the World Health Organization (WHO) protocol for fleas [18] and described elsewhere [16]. Briefly, a group of ten fleas and a strip of paper impregnated with technical grade insecticide (1.5 x 6 cm, Vector Control Research Unit, Penang, Malaysia) were placed in a tube. Each test was replicated at least four times for a total of 40 fleas minimum per insecticide bioassay. Negative controls consisted of two tubes of ten fleas each exposed to a clean filter paper. Mortality was recorded on test sheets at various time intervals for eight hours. Fleas unable to stand on their feet were considered dead or “knocked down”. After an exposure time of eight hours, insecticide treated papers were replaced with clean filter papers and fleas were kept in the tubes for 24 hours at 25°C and above 75% relative humidity. After 24 hours, the number of live and dead fleas was recorded. Mortality rates of ≥ 98% indicated susceptibility. A mortality rate of 80–98% indicted tolerance or suspected resistance, and < 80% indicated resistance [16]. For each collection, the name of the tested active ingredient, its respective concentration and the final mortality rate obtained is presented in Table 1. After testing, fleas were preserved in 70% ethanol.

**Table 1:** Collection location, number of fleas sequenced, and associated bioassay and genetic results for all 25 flea collections.

Collection	Insecticide And Dose of Exposure	Former Province/ District	Location	LAT	LON	Collection year	Months in Insectarium	Mort. Rate (bioassays)	# of Fleas (genetics)	L1014L Allele Freq.	L1014F Allele Freq.	L1014H Allele Freq.	Combined *kdr* Freq.	Citation
**Deltamethrin**														
1a	Delamethrin 0.05%	Antananarivo Renivohitra	Andranomanalina	-18.913	47.510	2002	216	1.00	33	1.00	0.00	0.00	0.00	16
2	Delamethrin 0.05%	Antananarivo Renivohitra	Tsenabe Isotry	-18.911	47.516	2005	8	0.34	38	0.14	0.66	0.20	0.86	16
3	Delamethrin 0.05%	Antananarivo Ankazobe	Andoharano	-18.319	47.116	2006	3.5	0.37	38	0.09	0.76	0.14	0.91	16
4	Delamethrin 0.05%	Antananarivo Betafo	Soavinarivo	-19.839	46.856	2006	Unknown	0.38	32	0.47	0.52	0.01	0.53	16
5	Delamethrin 0.05%	Antananarivo Miarinarivo	Miadamanjaka	-18.950	46.909	2006	4.5	0.33	39	0.00	0.99	0.01	1.00	16
6	Delamethrin 0.05%	Antananarivo Renivohitra	Ambodirano Ampefiloha	-18.924	47.505	2006	Unknown	0.89	35	0.26	0.73	0.01	0.74	16
7	Delamethrin 0.05%	Antananarivo Miandrarivo	Tsiroanomandidy	-18.771	46.051	2012	Unknown	0.59	29	0.00	0.91	0.09	1.00	n/a
8	Delamethrin 0.05%	Antananarivo Soavinandriana	Amparaky	-19.317	46.633	2012	Unknown	0.03	32	0.00	0.84	0.16	1.00	16
9a	Delamethrin 0.05%	Antananarivo Renivohitra	Ambohitrakely	-18.905	47.545	2017	Unknown	0.76	38	0.00	1.00	0.00	1.00	n/a
10	Delamethrin 0.05%	Antananarivo Renivohitra	Ambolokandrina	-18.922	47.560	2017	Unknown	0.11	35	0.06	0.94	0.00	0.94	n/a
11	Delamethrin 0.05%	Antananarivo Renivohitra	Andraisoro	-18.907	47.552	2017	Unknown	0.11	38	0.24	0.76	0.00	0.76	n/a
12	Delamethrin 0.05%	Fianarantsoa Ambositra	Ambanivolafotsy	-20.817	47.184	2006	1	0.45	20	0.03	0.95	0.03	0.98	16
13	Delamethrin 0.05%	Fianarantsoa Ambositra	Iarinoro Tsarasaotra	-20.533	47.245	2006	Unknown	0.90	39	0.00	1.00	0.00	1.00	16
14a	Delamethrin 0.05%	Fianarantsoa Farafangana	Farafangana prison	-23.027	47.787	2012	96	1.00	35	0.93	0.07	0.00	0.07	17
15	Deltamethrin 0.05%	Farafangana Fianarantsoa	Soanierana	-20.325	46.905	2013	5.5	0.28	40	0.00	1.00	0.00	1.00	n/a
16	Delamethrin 0.05%	Mahajanga I	Abattoir Mahajanga	-15.723	46.323	2005	5	0.55	31	0.02	0.98	0.00	0.98	n/a
17	Delamethrin 0.05%	Mahajanga I	Mahajanga prison	-15.729	46.320	2012	Unknown	0.42	31	0.16	0.84	0.00	0.84	17
18	Delamethrin 0.05%	Toamasina Ambatondrazaka	Ambohipananina	-17.826	48.417	2004	19	0.78	40	0.16	0.81	0.03	0.84	16
19	Delamethrin 0.05%	Toamasina Moramanga	Andaingo	-18.242	48.371	2004	Unknown	0.34	35	0.01	0.99	0.00	0.99	16
20	Delamethrin 0.05%	Toamasina Vavatenina	Vavatenina	-17.467	49.198	2007	Unknown	0.27	22	0.02	0.91	0.07	0.98	16
21	Delamethrin 0.05%	Toamasina Vavatenina	Vavatenina	-17.467	49.198	2020	2	0.10	206	0.00	1.00	0.00	1.00	n/a
22	Delamethrin 0.05%	Toliara Morombe	Morombe prison	-21.733	43.350	2012	9	0.50	40	0.11	0.89	0.00	0.89	17
**Other InsecticideInsecticides**														
1b	n/a	Antananarivo Renivohitra	Andranomanalina	-18.913	47.510	2002	132	n/a	29	0.33	0.67	0.00	0.67	16
9b	Permethrin 0.75%	Antananarivo Renivohitra	Ambohitrakely	-18.905	47.545	2017	Unknown	0.39	36	0.00	1.00	0.00	1	n/a
14b	n/a	Fianarantsoa Farafangana	Farafangana prison	-23.027	47.787	2012	24	n/a	2	0.25	0.75	0.00	0.75	17
23a	DDT 4.0%	Antananarivo Soavinandriana	Amparaky	-19.317	46.633	2004	112	0.13	40	0.00	0.89	0.11	1.00	14
23b	Permethrin 0.75%	Antananarivo Soavinandriana	Amparaky	-19.317	46.633	2004	112	0.91	43	0.00	0.85	0.15	1.00	14
24a	n/a	Antananarivo Renivohitra	Andranomanalina	-18.913	47.510	2005	1	n/a	9	0.06	0.83	0.11	0.94	16
24b	n/a	Antananarivo Renivohitra	Andranomanalina	-18.913	47.510	2005	96	n/a	30	0.00	1.00	0.00	1.00	n/a
25a	DDT 4.0%	Antananarivo Renivohitra	Antanimora prison	-18.914	47.542	2012	7	0.26	38	0.07	0.93	0.00	0.93	17
25b	Permethrin 0.75%	Antananarivo Renivohitra	Antanimora prison	-18.914	47.542	2012	7	0.32	38	0.00	1.00	0.00	1.00	17

Collections denoted with an "a" or "b" were collected from the same location and date but were bioassayed with different insecticides and/or at different time intervals. Collections were ordered by former province/district then by year of collection. Collections that were discussed in a previous paper are cited.

To investigate the VGSC mechanism of resistance to SPs in *X*. *cheopis*, we included flea collections from 1) geographically diverse locations in Madagascar representing 14 districts with various bioclimatic conditions, 2) urban neighborhoods in the capital Antananarivo, and 3) prisons (Fig 1). Our study primarily focused on fleas tested for resistance to deltamethrin because it has been the chosen insecticide for flea control in Madagascar since the 1990s. For three collections (9, 23, and 25), we also included fleas tested for resistance against two other insecticides (permethrin and/or DDT). Additionally, for three other collections (1, 14, and 24), we included fleas frozen at different time points (and, therefore, from different generations) to investigate the change in *kdr* frequencies in laboratory colonies over time in the absence of insecticide selection pressure. Maps were generated in R version 1.4.1106 [26] using the following packages: ggplot2, maptools, raster, rgdal, sp, rgeos, ggsn (Fig 1). Elevation data were provided by the Amazon Web Services Terrain Tiles and accessed through the package elevatr (zoom level 7).

### Molecular methods

In total, DNA was extracted from 1,395 *X*. *cheopis* fleas from Madagascar using the DNeasy Blood and Tissue (Qiagen, Hilden, Germany) and the Nucleospin DNA RapidLyse (Macherey-Nagel, Düren, Germany) kits. To determine the nucleotide sequence of the VGSC domain II segments 4–6 (S4-S6) in *X*. *cheopis* fleas, we created a multispecies gene alignment of publicly available mRNA and DNA sequences from GenBank to facilitate the design of forward primers (S1 Fig). Based on this alignment, we designed and tested several new forward primers located in the region that encodes residues 905–911 of domain II segment 4, based on the numbering of the *Musca domestica* VGSC protein (GenBank Accession AAB47604.1). We also modified a previously designed reverse primer [24] that encodes residues 1032–1038 by extending it four nucleotides upstream to increase specificity. We sequenced the section of the gene that encodes domain II S4-S6 of the VGSC protein using traditional Sanger sequencing methods. The target was amplified using forward primer DIIS4F4 and reverse primer A1ExtR, resulting in an amplicon length of 535 bp (Table 2).

**Table 2:** Primer and probe sequences used to amplify and sequence the section of genes that encodes for the domain II S4-S6 of the VGSC.

Primer/Probe Name	Assay Type	Sequence (5’ -> 3’)	Position on Alignment (S2 Fig)
DIIS4F4	Sanger—F	GCAAAATCTTGGCCGACTC	1bp-19bp
A1ExtR	Sanger/TaqMan—R	CGCTGTTGGAGCTGATAGACTC	514bp-535bp
IF1	Sanger/TaqMan—F	GCATTCCTTTCTTCTTGGCG	353bp-372bp
Probe1_CTT_FAM	TaqMan—Probe	TGGTTATTGGTAATCTTGTGG	377bp-397bp
Probe2_TTT_VIC	TaqMan—Probe	TGGTTATTGGTAATTTTGTGG	377bp-397bp
Probe3_CAT_NED	TaqMan—Probe	TGGTTATTGGTAATCATGTGG	377bp-397bp

Nucleotide positions are numbered to correspond to the *X*. *cheopis* voltage-gated sodium channel (VGSC) sequence (S2 Fig).

Polymerase chain reactions (PCRs) were carried out in 10 μL volumes, containing 2 μL of DNA template, 5.14 μL of UltraPure DNase/RNase-Free Distilled Water (Invitrogen), and the following reagents (given in final concentrations): 1 X PCR Buffer, 2.5 mM MgCl2, 0.2 mM dNTPs, 0.08 U Platinum Taq polymerase (Invitrogen, Carlsbad, CA, USA), and 0.4 μM of each primer. Targets were amplified according to the following thermocycler conditions: 96°C for 5 minutes, followed by 40 cycles of 95°C for 30 seconds, 60°C for 45 seconds, and 72°C for 30 seconds, ending with a final extension step of 72°C for 5 minutes. PCR products were then treated with ExoSAP-IT (Affymetrix, Santa Clara, CA, USA) to digest unused primers and dNTPs using 1 μL of ExoSAP-IT per 7 μL of PCR product under the following conditions: 37°C for 15 minutes, followed by 80°C for 15 minutes. Treated products were then diluted to 1/30 (or less, according to band intensity on an agarose gel) and sequenced in both directions using the same PCR primers in separate forward and reverse Sanger sequencing reactions with BigDye Terminator v3.1 Ready Reaction Mix (Applied Biosystems, Foster City, CA, USA). We used 10 μL volumes for sequencing reactions containing 5 μL diluted PCR product and the following reagents (given in final concentrations): 1 X Sequencing Buffer, 1 mM BigDye Terminator v3.1 Ready Reaction Mix, and 1 μM primer. The following thermocycler conditions were used: 96°C for 20 seconds followed by 30 cycles of 96°C for 10 seconds, 50°C for 5 seconds, and 60°C for 4 minutes.

Sanger sequencing reactions were run on a 16-capillary AB3130xl automated sequencer (Applied Biosystems, Foster City, CA, USA). The forward and reverse sequences for each flea were aligned into a single contig using SeqMan Pro from the Lasergene software package (DNASTAR, Madison, WI), and each sequence electropherogram was visually checked to ensure data quality. Heterozygous positions were called if both nucleotide peaks occupied the same base position in the electropherogram and had approximately the same peak heights. If insufficient signal was present for one of the possible bases, it was flagged as possible contamination and the flea was excluded from further analysis. Only ten samples had to be removed due to contamination. Any single nucleotide polymorphisms (SNPs) that were found only in one individual were confirmed by amplifying and sequencing the VGSC gene region a second time. As detailed in the results section, we discovered two non-synonymous SNPs in the first and second codon positions for the residue 1014 in domain II that both encode a different amino acid (L1014F and L1014H). We validated the allele sequences for a subset of four individuals by T-vector cloning and sequencing PCR amplicons from 18 individual clones.

Many of the fleas were stored in ethanol for long periods of time, leading to DNA degradation. To recover the sequence for these samples, we designed an internal primer (IF1), which amplified a 182 bp fragment that, when paired with the reverse primer A1ExtR (Table 2), still covered the *kdr* position. We used primers IF1 and A1ExtR for 314 fleas that failed to amplify in the full 535 bp assay and were able to recover 120 (38%). PCRs were carried out using all the same conditions as above except for the annealing temperature, which varied from 50–60°C depending on the sample. In total, we were not able to obtain sequences from 194 fleas.

To facilitate rapid genotyping of *kdr* mutations in *X*. *cheopis*, we developed a TaqMan real-time assay to discriminate among all three possible allele states that we discovered at codon position 1014 (L1014F/H). We made use of primers IF1 and A1ExtR from the 182 bp amplicon described above and designed new TaqMan probes (Table 2) using Primer Express v3.0 software (Applied Biosystems, Foster City, CA). PCRs were performed in 384-well optical plates and carried out in 10 μL volumes containing 1 μL of DNA template, 0.9 μL of UltraPure.DNase/RNase-Free Distilled Water, and the following reagents (given in final concentrations): 1 X TaqMan Environmental PCR Master Mix (Applied Biosystems), primers and probes, 0.35 M Betaine, and 0.25 U Platinum Taq Polymerase (Invitrogen). Primer concentrations used were 0.90 μM; Probe1_CTT_FAM and Probe3_CAT_NED were run at a final concentration of 0.15 μM, and Probe2_TTT_VIC at 0.30 μM. Thermocycling was conducted on a QuantStudio 12K Flex Real-Time PCR System (ThermoFisher Scientific) using the following conditions: 50°C for 2 minutes, 95°C for 10 minutes, followed by 45 cycles of 95°C for 15 seconds and 60°C for 1 minute. Genotyping calls were validated using fleas that had been sequenced, including 1,191 *X*. *cheopis* fleas from Madagascar as well as 45 *X*. *cheopi*s from Uganda and 51 *X*. *cheopis* from the US. Probe thresholds were set to 0.1 for analysis, and heterozygotes were only called if the two probe signals crossed the threshold within four PCR cycles (Cts) of each other.

### Statistical analyses

Statistical tests were performed in R version 1.4.1106 [26]. We used ordinary least squares regression (OLS) to test whether there was a linear relationship between the frequency of *kdr* mutations and the frequency of survivorship to 0.05% deltamethrin within flea collections. We assessed the normality of the residuals in the model that includes the frequency of either *kdr* mutation on percent survivorship using the Shapiro-Wilk test, which failed to reject the null hypothesis that the residuals are normally distributed (p = 0.705). For the purposes of this linear regression, we only included collections treated with deltamethrin that had 30 or more robust flea sequences to reduce the potential for bias caused by small sample sizes, which led to the exclusion of three collections (7, 12, and 20). We also fitted an exponential model using OLS by performing a square root transformation of the percent survivorship.

### Phylogenetic analysis

To assess the evolutionary history of susceptible and resistant alleles, we first needed to identify the specific VGSC alleles present in the Malagasy fleas. To do this, we started with the known alleles from homozygous sequences. Most heterozygotes resulted from combinations of the known alleles that we identified with Sanger sequencing. In a small number of heterozygotes, however, we needed to infer the haplotype phase because one allele was not represented in a homozygous sequence [27]. To make this process as conservative as possible, we inferred the smallest number of SNPs needed to explain each heterozygote sequence. For example, we assumed that an inferred allele different from a known allele by just one SNP was more likely than alternatives that required more SNPs to explain a heterozygous position in the Sanger sequence. Once we inferred the fewest number of SNPs and alleles to explain all the observed sequence types, we created a matrix of each possible allele pair. These pairings represented all the possible genotypes that we could have observed in our *X*. *cheopis* dataset. To determine the most likely allele combination that produced each observed heterozygous sequence, we used computational haplotype phasing [27]. This strategy relies on the Hardy-Weinberg principle that the frequencies of the two haplotypes observed in an individual are independent of one another. Therefore, the probability of observing a heterozygous individual with haplotypes *p* and *q* can be given by (*p* x *q*). Frequencies of the alleles were calculated from all fleas from Madagascar that had 1) robust sequence data for the full 535 bp amplicon, and 2) sequence types that were only explained by one allele combination. We then calculated the probability of observing each allele combination in question.

The possible evolutionary history of susceptible and resistant alleles was inferred using the Maximum Parsimony (MP) method in MEGA X [28]. To provide a broader context, we included additional fleas from lab colonies at the United States Centers for Disease Control and Prevention in Fort Collins, Colorado, which originated from *X*. *cheopis* populations collected in Uganda (*n* = 32) and the US state of Maryland (*n* = 51). We repeated the process previously described to sequence and determine the alleles present in these collections. We used all 535 nucleotide positions in the final dataset, of which 20 were informative. The MP tree was obtained using the Subtree-Pruning-Regrafting (SPR) algorithm (pg. 126 in ref. [29]) with search level 1 in which the initial trees were obtained by the random addition of sequences (100 replicates). For branches corresponding to partitions reproduced in at least 50% bootstrap replicates, the percentage of replicate trees in which the associated taxa clustered together in the bootstrap test (10,000 replicates) are shown next to the branches [30]. Evolutionary analyses were conducted in MEGA X [28]. We used these trees to broadly investigate the evolution and spread of *kdr* mutations in Madagascar.

## Results

### Phenotype testing

We found a high level of resistance to SPs in *X*. *cheopis* populations sampled throughout Madagascar. We focused on 25 flea collections from the IPM archives for which resistance phenotypes were evaluated with bioassays and adequate sample sizes (n ≥ 30) were available for genetic analysis. Of these, 22 collections had been tested for deltamethrin resistance, one collection (9b) was also tested for permethrin resistance, and another two collections (23 and 25) were tested for both permethrin and DDT resistance, totaling 27 phenotypic trials (Table 1). The bioassays demonstrated that only two of the 22 collections tested against 0.05% deltamethrin (1a and 14a) were susceptible (98–100% mortality). The average flea mortality rate in the remaining 20 non-susceptible collections was 42.6%. Two collections (6 and 13) were considered tolerant to 0.05% deltamethrin (80–97% mortality) and the remaining 18 collections were resistant (<80% mortality), as defined by WHO criteria [31]. The collections tested for permethrin and DDT resistance yielded average mortality rates of 53.7% and 19.4%, respectively.

### VGSC mutations

Consistent with the phenotypic results, we found a high frequency of *kdr* mutations. In total, we obtained robust VGSC sequences from 1,191 fleas. A mutation at the first position of the 1014 codon that results in an amino acid change (L1014F) at the *kdr* site in fleas [32,33] was found at a very high frequency in *X*. *cheopis* fleas from Madagascar (Table 1). The VGSC sequences were largely conserved; we found only 12 single nucleotide polymorphisms (SNPs) in the 535 bp sequence of the Malagasy fleas, which includes three exons and two introns. The introns (lower case letters in S2 Fig) contain four SNPs (nucleotide positions 152, 410, 412, and 423) found in approximately 5% of the Malagasy fleas sequenced, and two rare SNPs found in only one Malagasy flea (nucleotide positions 402 and 415). Four synonymous SNPs that occurred in exons at nucleotide positions 99, 231, 264, and 297 were found at the respective overall frequencies of 0.10, 0.05, 0.01, and 0.01 (underlined in S2 Fig) and do not alter any amino acids. However, two exon SNPs (nucleotide positions 391 and 392 in our PCR fragment; positions 3,117 bp–3,118 bp in the complete *Musca domestica* mRNA sequence; GenBank Accession KX431043.1) are non-synonymous in the first and second positions of the codon for amino acid position 1014 and alter the amino acid from leucine (L) to phenylalanine (F) or histidine (H), respectively. The L1014F mutation was previously described in *X*. *cheopis* from Uganda [24] and in China [25]. To our knowledge, we are reporting the first description of the second-position SNP (L1014H) in *X*. *cheopis*. The L1014F mutation has a much higher overall frequency (82%) in Madagascar than the L1014H mutation (4%). The L1014F mutation was found in all 25 collections, representing 14 districts in Madagascar. The low frequency L1014H mutation was also geographically widespread and was found in 12 collections from eight districts (Fig 1). Interestingly, we only found the L1014H mutation in collections where the L1014F mutation was also present. Nine of the collections that contained the L1014H mutation were located in the province of Antananarivo, but it was also found in a collection located in Fianarantsoa and two collections in Toamasina. Most fleas were homozygous for *kdr* mutations, and only 122 of 1,191 (10.2%) were heterozygotes that carried one *kdr* allele and one susceptible allele.

### Association of genotype and phenotype

**Fig 2:**
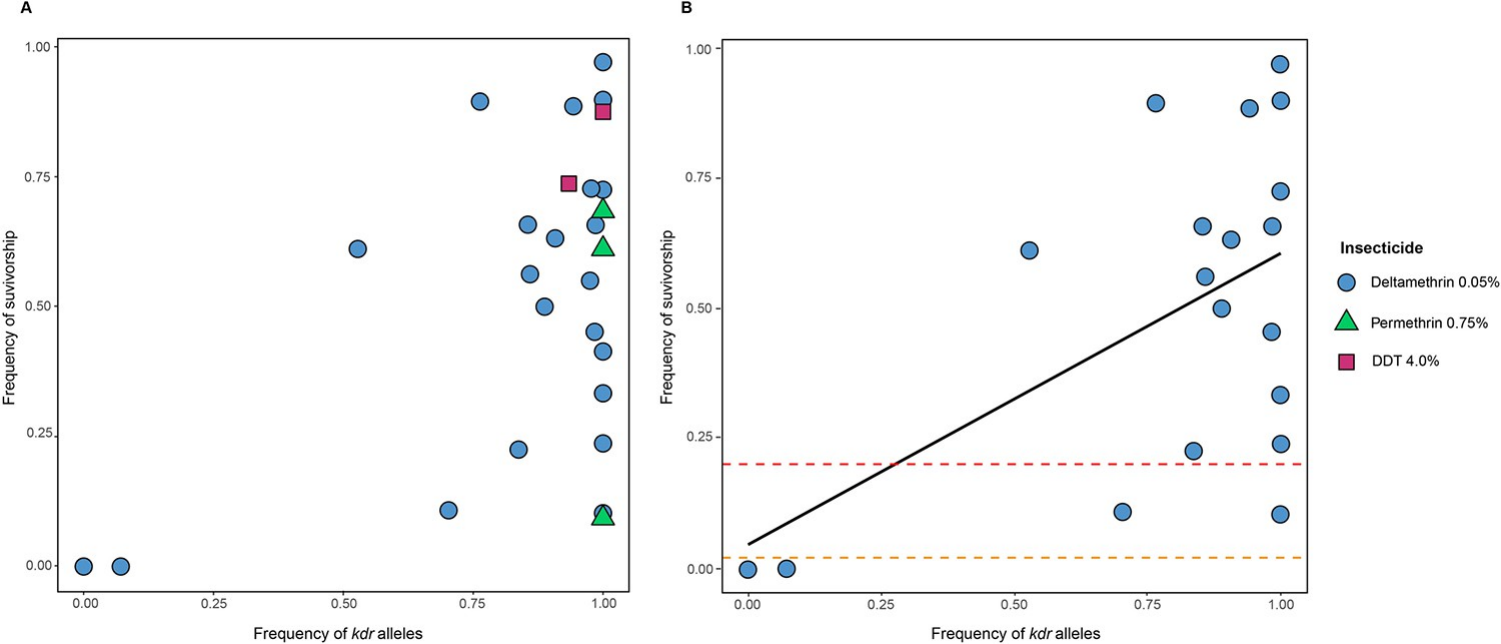
Relationship between frequency of *kdr* alleles and flea survivorship in collections of *Xenopsylla cheopis*. A) Relationship between frequency of L1014F/L1014H alleles in each population sample (collections; x-axis) and frequency of bioassay survivorship (y-axis) using 0.05% deltamethrin (22 collections), 0.75% permethrin (three collections), and 4.0% DDT (two collections). B) Same relationship as presented in A for only collections tested against 0.05% deltamethrin for which there were at least 30 robust sequences (19 collections). The orange dotted line separates flea collections that are susceptible (0–2% survivorship) and tolerant (2–20% survivorship) based on WHO criteria [31]. The red dotted line separates flea collections that are tolerant and resistant (>20% survivorship). A linear regression analysis revealed a significant relationship (*r* = 0.53, R^2^ = 0.28, p = 0.02).

We found a moderate correlation between the frequency of *kdr* alleles and the proportion of individuals that survived exposure to deltamethrin (Fig 2, Pearson’s *r* = 0.53, p = 0.02). Using a simple linear regression model, the frequency of both L1014F and L1014H alleles provides a significant prediction of flea survivorship to 0.05% deltamethrin (R^2^ = 0.28). We did not detect significant differences between the effects of the two *kdr* mutations on percent survivorship (t = 1.19, p = 0.25) so we combined the frequencies of L1014F and L1014H into a single explanatory variable, which we subsequently refer to as *kdr* frequency. The ability to detect these mutations in flea collections prior to insecticide treatment would be highly valuable for informing flea control efforts. Therefore, we developed a TaqMan PCR assay for the rapid detection of resistance mutations at the *kdr* codon. We found that this assay has a lower failure rate than Sanger sequencing (15% compared to 23%) and accurately genotyped 1,174 of the 1,201 (98%) fleas that had been previously sequenced. We further investigated the 27 fleas that had mismatched genotypes between the two methods and found that 12 were likely caused by contamination during the sequencing process and five by PCR error or contamination. For these 17 fleas, rerunning the TaqMan and/or sequencing assays from genomic DNA resulted in matching genotypes. The remaining ten fleas continued to produce conflicting results, potentially due to low DNA quality or cross-contamination of genomic DNA, and were excluded from further analysis.

### Phylogenetic analysis of VGSC alleles

In total, we identified seven known VGSC alleles (1–6, 10) and five inferred alleles (7–9, 11–12) in fleas from Madagascar, five of which carry the L1014F mutation, and two of which carry the L1014H mutation (Table 3). Alleles 1, 2, 3, 6, and 8 were identical to VGSC sequences previously reported for *X*. *cheopis* from China (haplotypes H1-F, H5-L, H2-F, H6-L and H4-L in that study, respectively) that were based on a shorter gene fragment (369 bp) [25]. The 12 alleles identified in Madagascar can create 66 possible sequence combinations, some of which could produce the same heterozygous nucleotide positions in a Sanger sequence through different allele combinations. Of the 22 sequence types found in the Malagasy fleas, six could have occurred through different allele combinations. All the allele combinations with the highest probability were also the most likely based on observed allele frequencies present in individual collections. The combined frequency of validated true alleles accounts for over 99% of the total dataset, thereby providing a high level of confidence in these alleles for phylogenetic analyses. Additionally, we identified four known VGSC alleles (13–16) and four inferred alleles (17–20) in fleas from Uganda, which created 28 possible sequence combinations. None of the observed sequence types could have occurred through alternative allele combinations. Lastly, we found two known VGSC alleles from Madagascar (alleles 5 and 10, Table 3) in fleas from Maryland.

**Table 3:** List of observed and inferred alleles used for phylogenetic analyses.

Allele #	Location	Estimated Frequency (w/in each country)	*kdr* genotype at the 1014 codon and nucleotides present at each of the 18 SNP sites																		
			AA1014 Nonsyn	99 Syn	152 Int	175 Int	178 Int	188 Int	231 Syn	264 Syn	297 Syn	402 Int	410 Int	411 Int	412 Int	418 Int	423 Int	425 Int	431 Int	434 Int	437 Int
1	Madagascar	0.8115	TTT	C	T	G	T	T	C	C	T	T	A	C	T	T	G	C	G	C	A
2	Madagascar	0.0747	CTT	T	T	G	T	T	C	C	T	T	A	C	T	T	G	C	G	C	A
3	Madagascar	0.0366	TTT	C	G	G	T	T	T	C	T	T	G	C	G	T	A	C	G	C	A
4	Madagascar	0.0288	CAT	T	T	G	T	T	C	C	T	T	A	C	T	T	G	C	G	C	A
5	Madagascar,	0.0347	CTT	C	T	G	T	T	C	C	T	T	A	C	T	T	G	C	G	C	A
	USA	0.2255																			
6	Madagascar	0.0098	CTT	C	G	G	T	T	T	T	C	T	A	C	T	T	G	C	G	C	A
7	Madagascar	0.0010	TTT	T	T	G	T	T	C	C	T	T	A	C	T	T	G	C	G	C	A
8	Madagascar	0.0005	CTT	C	G	G	T	T	T	C	T	T	G	C	G	T	A	C	G	C	A
9	Madagascar	0.0005	TTT	C	G	G	T	T	T	T	C	T	A	C	T	T	G	C	G	C	A
10	Madagascar,	0.0005	CTT	C	T	G	T	T	C	C	T	C	A	C	T	T	G	C	G	C	A
	USA	0.7745																			
11	Madagascar	0.0010	TTT	C	G	G	T	T	C	C	T	T	G	C	G	T	A	C	G	C	A
12	Madagascar	0.0005	CAT	C	T	G	T	T	C	C	T	T	A	C	T	T	G	C	G	C	A
13	Uganda	0.3750	CTT	C	T	A	A	T	T	C	T	T	G	C	G	T	A	C	G	C	G
14	Uganda	0.2344	CTT	C	T	G	T	T	T	C	T	T	A	C	G	T	A	G	A	A	A
15	Uganda	0.0938	CTT	C	G	G	T	T	T	C	T	T	G	C	G	T	A	C	G	C	G
16	Uganda	0.0313	CTT	C	G	G	T	T	T	C	T	T	A	C	G	T	G	C	G	C	A
17	Uganda	0.0938	CTT	C	T	G	T	C	T	C	T	T	A	C	G	T	A	G	A	A	A
18	Uganda	0.1406	CTT	C	T	G	T	C	T	C	T	T	A	T	G	A	G	C	A	A	A
19	Uganda	0.0156	CTT	C	T	A	A	T	T	C	T	T	A	C	G	T	A	G	A	A	A
20	Uganda	0.0156	CTT	C	T	A	A	T	T	C	T	T	A	T	G	A	G	C	A	A	A

We found 20 parsimony-informative polymorphic sites in the 535 bp segment of the VGSC gene from *Xenopsylla cheopis*; 11 sites in the flea sequences from Madagascar (alleles 1–12), 12 sites in the flea sequences from Uganda of which nine were unique (alleles 13–20). The SNPs associated with each allele type are shown. SNP positions are numbered to correspond to the *X*. *cheopis* VGSC sequence (S2 Fig). Estimated allele frequencies within each country are calculated from all fleas with robust sequences across all SNP sites. For the 18 SNP sites that do not result in an amino acid change, synonymous (Syn) and intronic (Int) SNPs are labeled. GenBank accession numbers are OP508035-OP08054.

**Fig 3:**
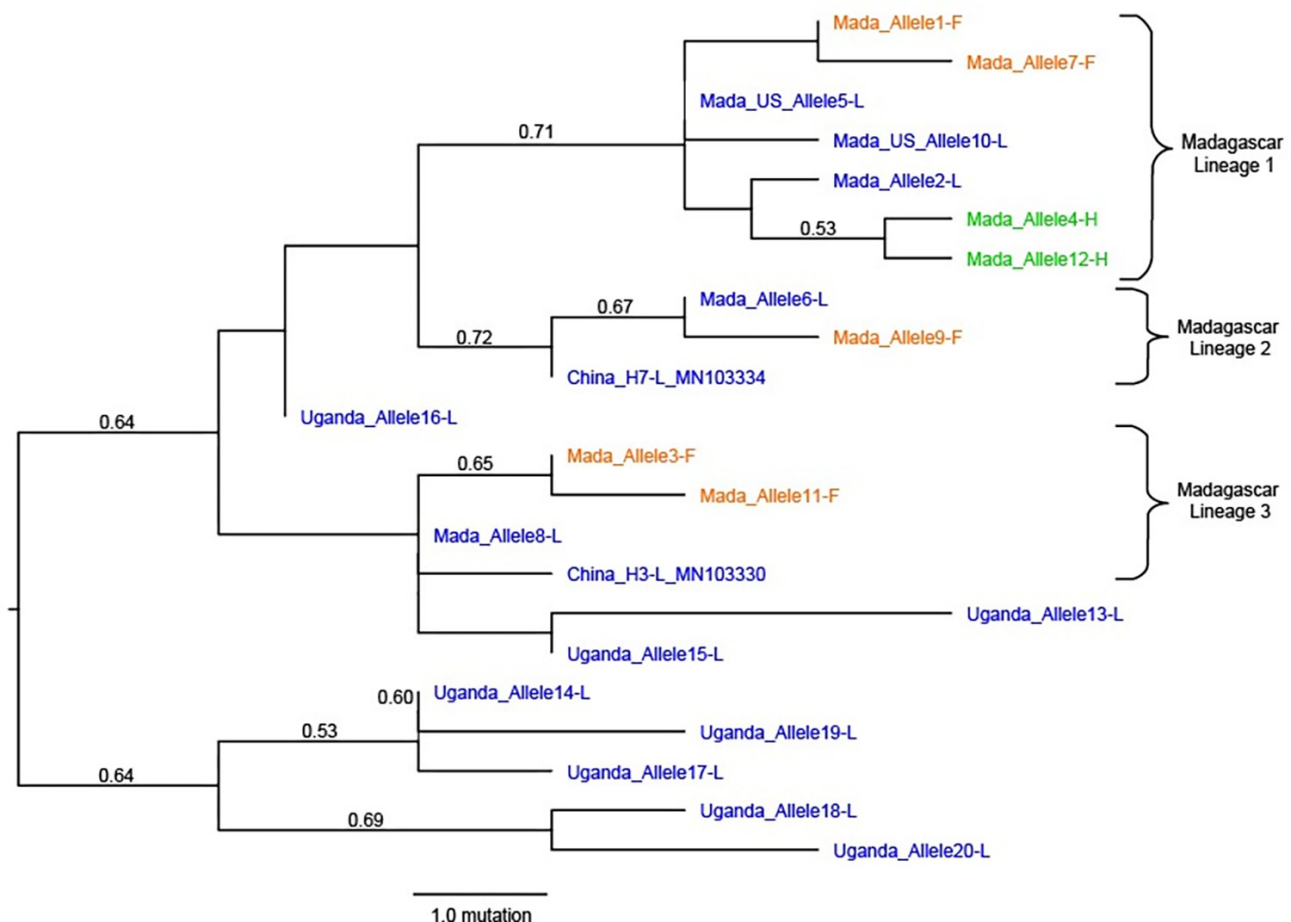
Phylogenetic analysis of voltage-gated sodium channel (VGSC) alleles in *Xenopsylla cheopis* fleas from Madagascar, Uganda, US, and China. Alleles (Table 3) containing the L1014L, L1014F, and L1014H genotypes are shown in blue, orange, and green, respectively. Branch labels correspond to bootstrap values based 10,000 replicates; values less than 0.50 are not shown. The tree is rooted on the lineage containing Uganda alleles 14, 17, 18, 19, and 20. Sequences China_H7-L_MN103334 and China_H3-L_MN103330 were pulled from NCBI Genbank entries with accession numbers MN103334.1 and MN103330.1, respectively (21).

Tree #1 out of the 70 most parsimonious trees is shown (Fig 3). The consistency index was 0.58, the retention index was 0.78, and the composite index was 0.46 for all sites. The phylogenetic tree reconstructed from these DNA sequences revealed that the 12 Malagasy alleles clustered into three main lineages, each containing susceptible alleles with the ancestral genotype (L1014L) and the common *kdr*-resistant genotype (L1014F). The less common L1014H mutation is only found in Malagasy lineage 1. Additionally, within lineage 1, it appears that alleles containing *kdr* mutations form two distinct clusters. From this phylogenetic analysis, we infer that resistant allele L1014F arose independently at least three times in Madagascar. The L1014H mutation was yet another independent event that most likely arose in Madagascar. The Uganda alleles (13–20) primarily formed one distinct lineage, with three alleles falling within the Malagasy lineages. Both US alleles (5 and 10) fell within the Malagasy lineage 1.

### Potential fitness cost of *kdr* mutations

To better understand the possibility that *kdr* alleles may carry a fitness cost, we compared fleas from three flea colonies (1, 14, and 24) at two time points after collection. At 11 years after collection, collection 1 had a L1014F frequency of 0.67, which then dropped to zero seven years later (18 years after collection). Collection 14 had a L1014F frequency of 0.75 two years after collection, which decreased to 0.07 eight years after collection. Collection 24 had L1014F and L1014H frequencies of 0.83 and 0.11 one month after collection, respectively. Eight years after collection, the L1014F frequency increased and became fixed at 1.00, whereas L1014H and the susceptible genotype had both dropped to zero by the second time point (Table 4). We emphasize that none of these lab colonies had been maintained under insecticide selection pressure in the IPM insectarium.

**Table 4:** Frequencies of *kdr* allele states in three *Xenopsylla cheopis* laboratory colonies changed through time despite the absence of selection pressure from deltamethrin.

Collection	# of Fleas	Years in Insectarium	L1014L Allele Freq.	L1014F Allele Freq.	L1014H Allele Freq.
1b 1a	29	11	0.33	0.67	0.00
	33	18	1.00	0.00	0.00
14b 14a	2	2	0.25	0.75	0.00
	35	8	0.93	0.07	0.00
24a 24b	9	0	0.06	0.83	0.11
	30	8	0.00	1.00	0.00

Collections refer to the same populations of fleas shown in Table 1 Fleas were reared in the insectarium without exposure to any insecticide.

## Discussion

Deltamethrin resistance is widespread in *X*. *cheopis* populations from Madagascar. Approximately 78% of the fleas tested against deltamethrin and utilized in this study were considered resistant based on criteria defined by WHO [31]. These resistant fleas were collected across 14 districts and across 18 years, suggesting resistance to SPs is extensive in Madagascar and has been present for at least the past two decades. These data are supported by previous reports of resistance of fleas to deltamethrin, which was first described in 1996 [34]. Recent studies have found that 80–100% of tested flea populations collected throughout Madagascar have moderate to high levels of resistance to deltamethrin, presenting a critical problem for flea control efforts [14,16].

Knockdown mutations are present at high frequencies in *X*. *cheopis* collected throughout Madagascar. The average frequency of *kdr* mutations in collections tested against deltamethrin is 0.83. Our results found that there is a positive linear relationship between the frequency of *kdr* resistance genotypes and population levels of phenotypic resistance to deltamethrin based on bioassay results for each collection (Fig 2, *r* = 0.53, p = 0.02). Studies in other insects also report an association between SP resistance and the frequency of *kdr* alleles [35–37]. Jamroz et al. (1998) found that super-resistant horn fly populations that encountered weekly treatments of cyhalothrin in the laboratory had a *kdr* allele frequency of 0.98, whereas less resistant and susceptible populations had *kdr* allele frequencies of 0.66 and 0.00, respectively [38]. The frequency of *kdr* mutations in the mosquito *Anopheles gambiae* is significantly correlated with resistance to deltamethrin, permethrin, and DDT [39]. An increase in *kdr* alleles was also observed in *A*. *gambiae* after the initiation of an indoor residual spraying (IRS) program. The *kdr* mutation was initially found in 50% of the included mosquitoes and explains the initial failure of SPs to reduce mosquito populations [40]. It is critical to note that *kdr* allele frequencies do not have to be particularly large to cause the failure of a vector control program.

Although we found a strong association between the survivorship of flea populations to deltamethrin exposure and the frequency of *kdr* mutations, this variable only explains about 28% of the variation observed in the bioassay data and there are several possible explanations for this variability. First, metabolic detoxification is another significant alternative mechanism of SP resistance in many insect species, and this could have increased the survival of both susceptible and resistant genotypes beyond the expected level from the regression line. In mosquitoes, the frequency of mutations in the VGSC and activity of glutathione S-transferases facilitates resistance to SPs [41]. Additionally, *X*. *cheopis* collected from locations in Uganda with a history of IRS had significantly higher expression levels of alpha- and beta-esterases than fleas collected from locations without a history of IRS [24]. This suggests that increased expression of these enzymes also may play a role in enhancing the insect tolerance to insecticides, and this mechanism could be investigated in Madagascar populations using bioassays that include synergists to enhance the action of the active ingredient. Second, other mutations in the VGSC gene that have yet to be sequenced may play a role in SP resistance, but these remain undescribed in *X*. *cheopis* from Madagascar. For example, the F1534S mutation in Domain III Segment 6, which was not covered by our 535 bp amplicon, is correlated with SP resistance in mosquitoes [42]. Third, bioassay results can vary based on several factors, including the type of assay used. Specifically, studies that compared the CDC bottle assay [43] and the standardized WHO tube test [18] in mosquitoes found that the results differed [44,45]. The standard WHO bioassay protocol and diagnostic insecticide concentrations for fleas were adapted from the WHO bioassay protocol designed for adult mosquitoes, a flying insect, and could present problems regarding appropriate dosage and nonconstant exposure of fleas to the insecticide. Inconsistent exposure could potentially lead to higher or lower survival than expected, depending on the final exposure for each individual flea. Confounding stochastic effects could also result from poor flea body condition (described below in the limitations to this study). Lower dosages and the use of a Petri dish instead of a tube may provide more accurate susceptibility data on *X*. *cheopis* [24]. Additionally, it is possible to topically apply insecticide to adult fleas, providing more precise dose response information [46]. Despite these caveats, substantial evidence suggests that both L1014F and L1014H mutations play an important role in conferring resistance to SPs [21,47–50]. Future studies are needed to further explore the role of metabolic detoxification in *X*. *cheopis* fleas that are resistant to SPs. Additionally, it is pertinent to reevaluate the widely used WHO protocol for fleas to ensure that bioassay results for fleas are consistent and accurate.

**Fig 4:**
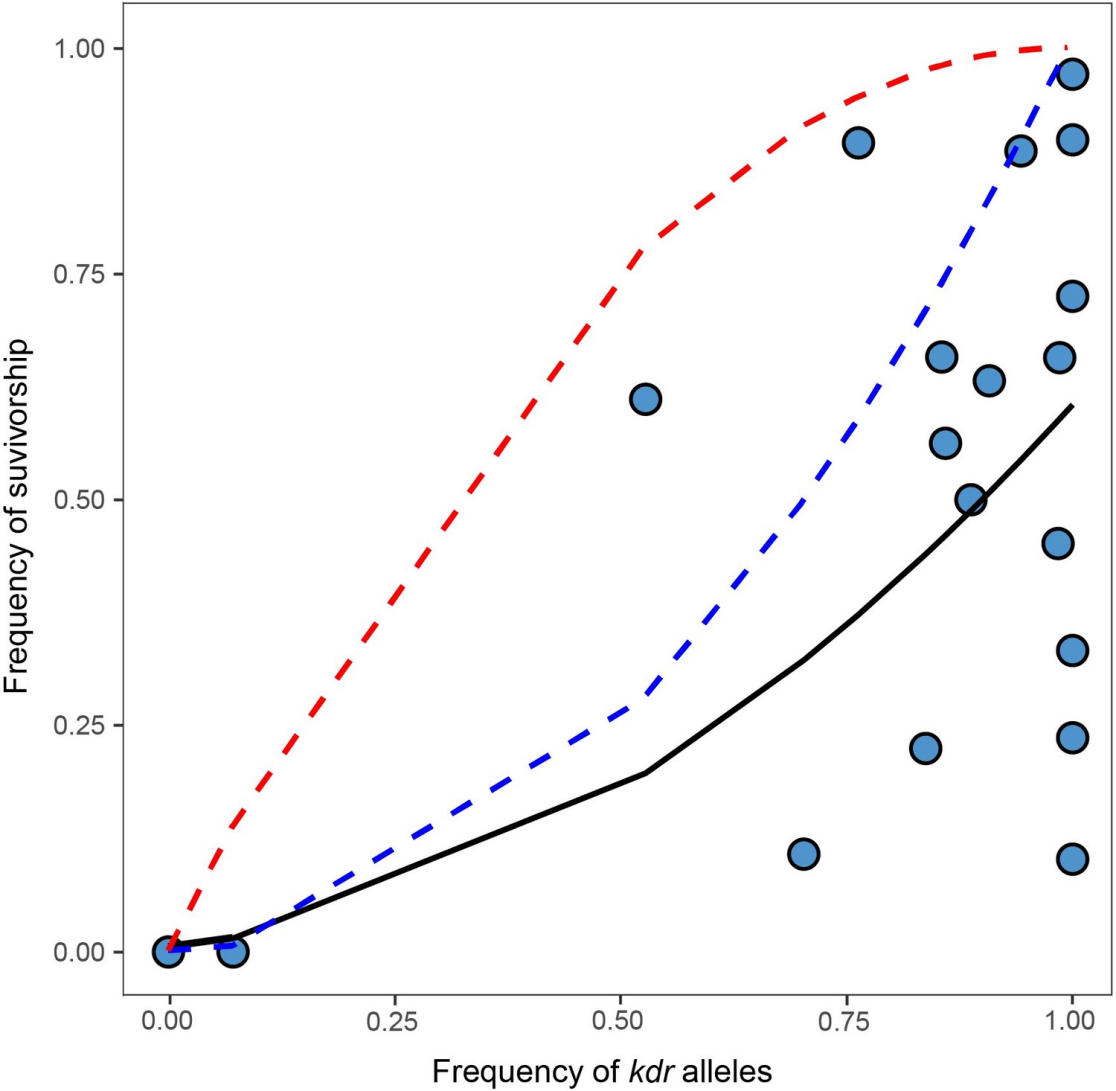
An exponential regression model supports the possibility that *kdr* resistance is recessive in *Xenopsylla cheopis*. There is a significant linear relationship (*r* = 0.70, R^2^ = 0.49, p = 0.00084) between the frequency of *kdr* alleles (x-axis) and the square root of the frequency of survivorship to 0.05% deltamethrin (y-axis) in 19 *X*. *cheopis* collections that had at least 30 robust sequences, which explains the observed data better than the linear relationship in Fig 2 (*r* = 0.53, R^2^ = 0.28, p = 0.02). The solid black line shows the regression line for the observed data; the blue dotted line shows the theoretical relationship between the frequency of *kdr* alleles and percent survivorship under the assumptions that *kdr* is recessive and only fleas homozygous for the *kdr* resistance mutation would survive exposure to deltamethrin; the red dotted line shows the theoretical relationship assuming *kdr* is dominant.

Studies on *kdr* inheritance patterns in some insect species suggest that *kdr* is a recessive trait. It is unknown whether *kdr* is also recessive in *X*. *cheopis*. If so, an exponential model would be more appropriate for explaining the observed data than a linear regression model, which implicitly assumes that *kdr* is a codominant trait (*i*.*e*., that heterozygotes express both the susceptible and resistant alleles). If *kdr* was a recessive trait, we would expect an exponential relationship between the frequency of *kdr* alleles and percent survivorship of flea collections because a single copy of the *kdr* mutation would not confer resistance and only homozygous *kdr* fleas would survive exposure to deltamethrin. Accordingly, our exponential model explained more of the variability observed in the bioassay data than the linear relationship model (*r* = 0.70, R^2^ = 0.49, p = 0.0008, Fig 4). We found 122 fleas heterozygous for a *kdr* mutation in the 1,191 sequenced fleas (0.102). Among 104 of these heterozygotes tested against 0.05% deltamethrin, 71 died after exposure to 0.05% deltamethrin whereas 33 survived. When individual collections were considered, a similar pattern was observed in 12 of the 14 collections with heterozygous fleas. Together, these results are consistent with the possibility that *kdr* mutations in fleas are recessive. In *A*. *gambiae*, permethrin, deltamethrin, or DDT exposure appears to affect heterozygous mosquitoes in the same way as homozygous susceptible individuals, whereas resistance is mainly observed in L1014F homozygotes [51,52]. In *M*. *domestica*, L1014F appears to be incompletely recessive, with some heterozygotes that survive, whereas L1014H appears to be incompletely dominant (heterozygotes and *kdr* homozygotes had similar survivorship rates) [53]. Future studies will be needed to confirm the hypothesis that *kdr* is recessive in *X*. *cheopis*.

The rise of genetic resistance due to the extensive use of deltamethrin may generate cross-resistance to other insecticides that use the same mechanisms. Flea collections in Madagascar also exhibited resistance to permethrin, a related SP, as well as DDT, which has not been used for vector control in Madagascar for decades. Although DDT is an organochloride insecticide, it is similar to SPs in function. Namely, the preferential binding of these insecticides to the open conformation of insect sodium channels leads to prolonged channel opening, causing a state of hyperexcitability that eventually leads to paralysis and death [54]. The ability of *kdr* mutations to confer resistance to both SPs and DDT has been documented in many insect species [39,55,56] and suggests that these mutations act by a general mechanism that is not inhibited by small differences in insecticide binding properties. Indeed, attempts to model the open state conformation of the VGSC protein demonstrate that the *kdr* mutations are not located in the predicted insecticide binding pocket but, instead, are located near the domain III gating-hinge, which is important for conformational change of the protein [22]. Thus, the *kdr* mutations may cause resistance by reducing the availability of open-state channels and decreasing the binding affinity of SPs and DDT to open channels [21–23]. The L1014F mutation leads to insensitivity to deltamethrin in *Drosophila melanogaster* through this mechanism, reducing the binding affinity of the insecticide to open channels by 20-fold and increasing the rate of disassociation of the drug once bound to an open channel [48]. Additionally, different *kdr* mutations can confer varied cross-resistance profiles to SPs and DDT. For example, the L1014F mutation confers relatively equal amounts of resistance to deltamethrin, permethrin, and DDT, whereas the L1014H mutation is more effective against deltamethrin and less effective against permethrin and DDT when expressed in *Xenopus* oocytes [50]. This could have interesting implications for the alternative fitness of *kdr* mutations when certain insecticides are being used. Although the L1014H mutation is more effective against deltamethrin, it is rare in *X*. *cheopis* collections in Madagascar, which could possibly indicate that it is a relatively recent mutation. The finding that the *kdr* mutation can cause resistance to many insecticides raises concerns about the overall efficacy of SPs for controlling fleas in Madagascar.

A growing number of studies have demonstrated that insecticide resistance carries a fitness cost ranging from behavioral to physiological effects (reviewed in [57]). Interestingly, collections 1 (collected in 2002) and 14 (collected in 2012) were initially resistant to deltamethrin, with average mortality rates of approximately 47% and 44%, respectively [16,17]. These flea collections continued to be reared in the IPM insectarium in the total absence of insecticide selection pressure, and bioassay testing against deltamethrin in 2019 revealed 100% mortality rates, suggesting they reverted to complete susceptibility within 7–17 years. Genotyping samples obtained in 2020 from these collections demonstrated that the frequency of *kdr* mutations had become very low (0.07% in collection 14a) or completely absent (collection 1a). Retrospective genotyping of fleas from these collections revealed that the *kdr* frequency had been much greater in the past (0.67 and 0.75, respectively, Table 4). The decrease in *kdr* frequency over time in these two collections could suggest that *kdr* mutations have a fitness cost. Only one lab colony in our study (collection 24) showed an increase in *kdr* frequency over time, from 0.83 to 1.00 in samples separated by eight years (Table 4). It is possible that genetic drift acting on a small population could be responsible for the fixation of the *kdr* allele in this case. A fitness cost has been hypothesized as a mechanism to explain the reversion to susceptible phenotypes that has been documented in populations of other insects [58–60]. Additionally, several studies report a decrease in *kdr* alleles of insect populations in the absence of insecticide selection pressure [35,61–63]. In *A*. *aegypti*, mosquitoes carrying the *kdr* mutations demonstrated slower development, decreased fecundity, and affected behavior [61]. Because of these fitness costs, in the absence of insecticide selection pressure we would expect that resistance mutations can be lost from a population over time; therefore, refraining from the use of SPs might eventually restore their effectiveness. However, *kdr* frequency increased from 0.83 to 1.00 after eight years in collection 24, producing conflicting results. If this colony experienced periods of small population size, genetic drift may have led to the fixation of the *kdr* allele. Additional studies that measure changes in *kdr* frequencies in *X*. *cheopis* over time in the absence of insecticide selection pressures are needed.

**Fig 5:**
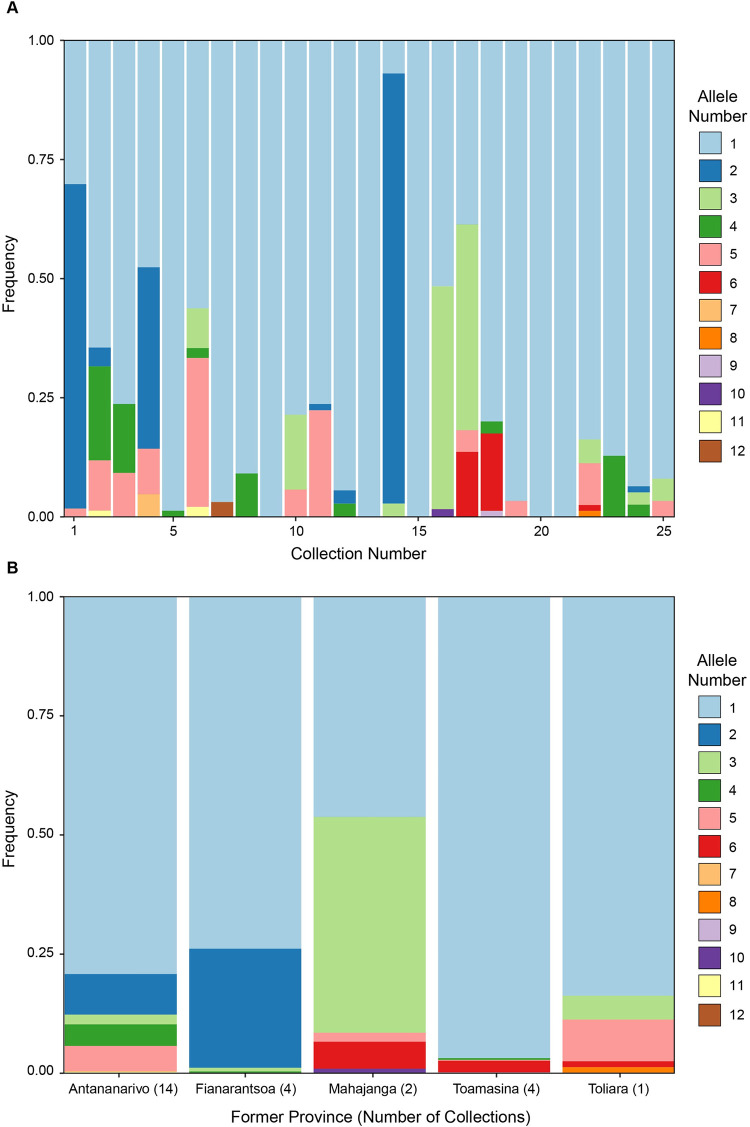
Allele frequencies across geographic locations of Madagascar. a) Frequency of the 12 alleles (Table 3) in each of the 25 flea collections. b) Frequency of the 12 alleles across the five former provinces of Madagascar. Allele 1 was the most frequent and widespread allele in our dataset.

Our phylogenetic analyses suggest that the *kdr* mutations independently arose within three major allele lineages in Madagascar (Fig 3), similar to the evolutionary scenario described for *X*. *cheopis* in China [25]. Assuming that susceptibility was an ancestral state within each major lineage, the common resistance SNP (L1014F) appears to have evolved at least three times, thus suggesting convergent evolution driven by selection pressure from deltamethrin. Furthermore, the intense insecticide selection pressure in Madagascar has given rise to a second, rare mutation (L1014H) in lineage 1. The Malagasy *kdr* alleles 1 and 3 are identical to *kdr* haplotypes H1 and H2 from Chinese populations, which means it is possible the L1014F mutation in lineages 1 and 3 could have originated in China. However, *kdr* evolution is a nearly ubiquitous outcome for insect populations that are under long-term selective pressure from SPs; therefore, it is reasonable to predict that these mutations arose independently in Madagascar after *X*. *cheopis* had colonized the island, which happened long before SPs were developed. In Madagascar, both L1014F and L1014H probably evolved in local populations at first and then, over time, spread widely across the island. This scenario would explain the presence of multiple distinct lineages at many sampling locations, each with at least one *kdr* allele, and the overall lack of geographic stratification within each lineage (Fig 5). However, alleles of lineage 2 (alleles 6 and 9) are not found in Antananarivo, whereas alleles 7, 11, and 12 are only found in Antananarivo. Additionally, alleles 8, 9, and 10 were only found in one collection each from Toliara, Toamasina, and Mahajanga. It is possible that these alleles went extinct shortly after emerging in the population or have not had enough time to spread across Madagascar. Although other explanations for the evolution of *kdr* mutations are possible, the most likely scenario is consistent with convergent evolution occurring due to the presence of heavy selection pressure by deltamethrin.

The absence of location-specific lineages indicates that gene flow between geographically diverse flea populations in Madagascar is high (Fig 5). Gene flow between flea populations is most likely facilitated by the movement of their primary rodent host, the black rat (*Rattus rattus*). Genetic analyses of the population structure of black rats in Madagascar demonstrate that populations are separated into only two main groups: northern Madagascar and the rest of the island. In addition, population structure estimates using microsatellite markers are very low [64], suggesting that gene flow among rat populations is high and that geographic distance does not seem to differentiate populations. Flea populations are slightly more structured in Madagascar, differentiating into three main clusters with evidence of gene flow between clusters [65]. Furthermore, selection pressure from insecticides probably occurs in many flea populations outside of the plague-endemic central highlands. For example, collections 14, 17, and 22 were sampled from coastal prisons where SPs are used extensively to control various insect pests [14,17]. SPs are also part of commonly used household products to control insect pests. Because malaria is endemic, IRS is commonly applied to reduce anopheline mosquito populations. Insecticide-treated nets (ITNs) are also widely used to protect against anopheline mosquito bites; both IRS and ITN methods use SPs. Insecticide resistance in fleas from treated houses, prisons, and plague endemic regions could select for the *kdr* mutation, which is likely under enough selection pressure for *kdr* mutations to be maintained even in areas where plague is not endemic.

There is currently no effective vaccine available to prevent plague infection, making insecticides the primary tool for reducing the incidence of human plague in addition to antimicrobial treatment. The widespread presence of resistance and *kdr* mutations indicates that deltamethrin will not effectively reduce many flea populations in Madagascar. Because *kdr* mutations are shown to confer resistance to many types of SPs and DDT, it is important that flea control efforts utilize an insecticide that kills the insect via an alternative mechanism. The strategy of switching between insecticides that utilize different mechanisms can be an effective way to control vector populations long-term [66]. Specifically, switching from DDT/pyrethroids to carbamates effectively reduced the incidence of mosquitoes and malaria in parts of Africa [40,67]. In response to concerns about SP resistance, the National Plague Control Program of Madagascar now routinely uses fenitrothion, an organophosphate, to control fleas. It is unclear how often programs should switch between insecticides. However, this study demonstrated that resistance reversion may have occurred completely after 7–17 years, but this will likely depend on how quickly resistance is lost in the absence of insecticide selection pressure. Understanding the fitness cost of resistance, both at the phenotypic and genetic level, will provide critical insight into the most effective strategies for insecticide use on disease vectors. Additionally, the use of accurate dosage for insecticide application will be important for effective flea control, and insecticide dusting may only provide residual and short-term exposure to the chemical. The use of edible baits containing insecticides provides continual, focused exposure of fleas to the insecticide via feeding on the host and is effective at reducing flea abundance on black-tailed prairie dogs [68]. However, the feasibility of this technique for commensal rodents such as *R*. *rattus*, the principal plague reservoir and host for *X*. *cheopis* in Madagascar, needs more investigation. Another suggested alternative is bait boxes, which attract rodents into boxes filled with insecticide using baits with the goal of thoroughly and directly exposing the fleas to the insecticide [69]. Unfortunately, this method appears to be ineffective at reducing indoor *X*. *cheopis* in Madagascar [70]. Additional research on alternative strategies will be important for the long-term prevention of plague.

There are several limitations to this study to note. First, bioassays require many healthy fleas for accuracy. Fleas collected from the field were transported to the lab, which is often a long journey from rural areas. Sometimes very few of the fleas survived transportation, reducing the number of parents available to establish the colony. Because of this, survivors that were collected from the field needed to be bred until a large, healthy colony was obtained. Consequently, the fleas the bioassays were performed on may be several generations removed from the fleas collected in the field. Second, sampling a limited number of fleas from a field population represents a genetic bottleneck. It is possible that some alleles present in the population were not represented in the sample. Further, if the sample is small enough, genetic drift could cause the loss and fixation of alleles in the established colony. Third, most of the fleas were collected during epidemic response actions and, thus, after insecticide treatment failed. Therefore, the fleas collected have likely already gone through a major selection event, inflating the number of resistant individuals. For these reasons, the data presented in this study are likely not completely representative of the phenotypes and genotypes of flea populations in the field. Despite these caveats, this study provides the most comprehensive understanding of the prevalence of SP mutations in Malagasy flea populations thus far.

The use of molecular tools to track the frequency of *kdr* mutations provides an accurate prediction of the overall susceptibility of insects to deltamethrin and other SPs. Although this mutation is likely not the only genetic mechanism involved in conferring resistance to SPs, it can be used to quickly identify populations where resistance may pose problems for vector control. To facilitate the rapid and accurate genotyping of *X*. *cheopis* for *kdr* mutations, we developed a TaqMan assay that is capable of distinguishing between the three possible genotypes with a single PCR assay. We propose that this genotype information can be used in addition to or in place of bioassay data to determine the susceptibility status of fleas to SPs. In this way, the TaqMan assay would serve as a useful complement to bioassays, because it can be used on a greater number of field collections and does not require live fleas. It can be used to rapidly genotype a large number of individual fleas (30 or more) from each collection to estimate the proportion of individuals expected to survive treatment with deltamethrin. This assay accurately genotyped *X*. *cheopis* from Madagascar, Uganda, and the US, making it an important tool for the surveillance of resistance to SPs worldwide.

## Supporting information

S1 Fig Multi-species alignment of a segment of domain II of the voltage-gated sodium channel (VGSC) gene. This alignment includes residues 899–917 (numbered according to the *Musca domestica* amino acid sequence, GenBank Accession AAB47604.1). GenBank accession numbers are listed after the species name and nucleotide sequence type. Coded amino acids are represented above the nucleotide sequence.
(TIF)

S2 Fig Nucleotide sequence of the domain II S4-S6 region of the *Xenopsylla cheopis* voltage-gated sodium channel (VGSC) gene. Mutations are numbered according to the *Musca domestica* amino acid sequence (GenBank Accession AAB47604.1). Coded amino acids are represented above the nucleotide sequence, exons are represented as capital letters, and introns are represented as lower case letters. Single nucleotide polymorphisms (SNPs) are underlined. The boxed sites 391 and 392 correspond to the *kdr* L1014F/H mutation. Primer and probe sequences are shown in bold font.
(TIF)
