# Supplementary figures and images for: Knockdown resistance mutations are common and widely distributed in Xenopsylla cheopis fleas that transmit plague in Madagascar
Source: PLoS Negl Trop Dis. 2023 Aug 22;17(8):e0011401. doi: 10.1371/journal.pntd.0011401 (PMC10443838; doi:10.1371/journal.pntd.0011401)

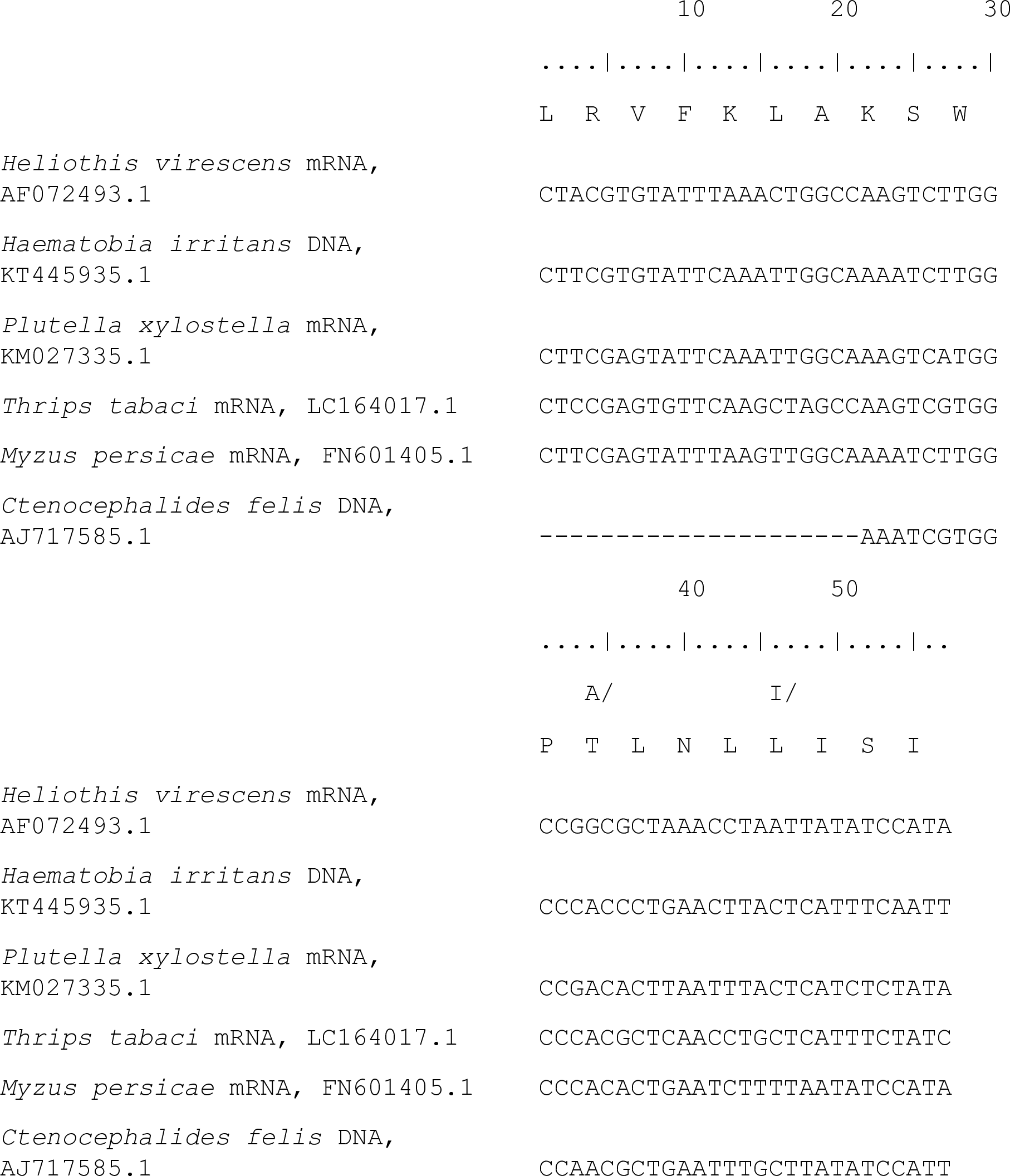

Supplement: S1 Fig — This alignment includes residues 899–917 (numbered according to the Musca domestica amino acid sequence, GenBank Accession AAB47604.1). GenBank accession numbers are listed after the species name and nucleotide sequence type. Coded amino acids are represented above the nucleotide sequence. (TIF) [file pntd.0011401.s001.tif]

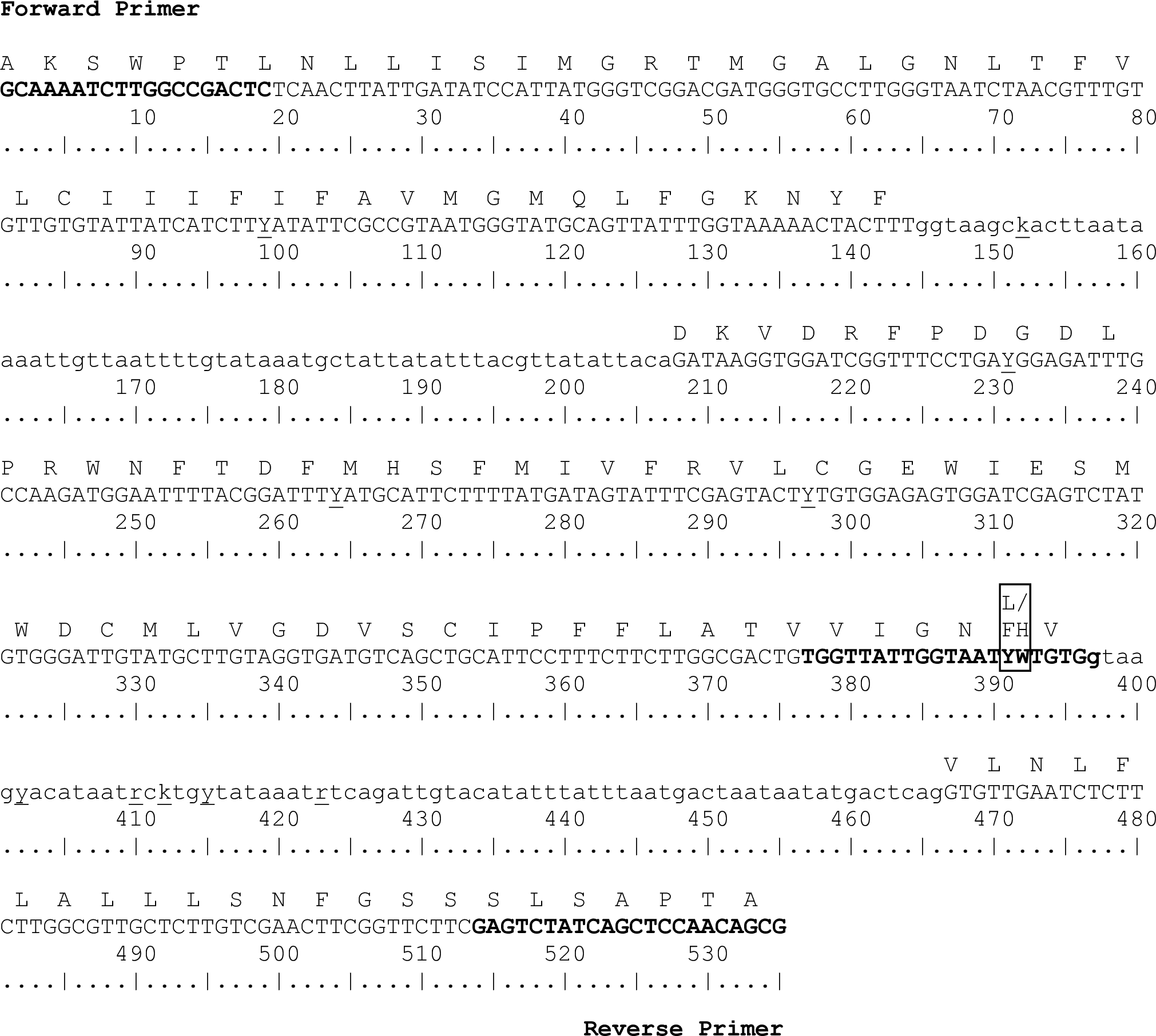

Supplement: S2 Fig — Mutations are numbered according to the Musca domestica amino acid sequence (GenBank Accession AAB47604.1). Coded amino acids are represented above the nucleotide sequence, exons are represented as capital letters, and introns are represented as lower case letters. Single nucleotide polymorphisms (SNPs) are underlined. The boxed sites 391 and 392 correspond to the kdr L1014F/H mutation. Primer and probe sequences are shown in bold font. (TIF) [file pntd.0011401.s002.tif]
